# RNA folding with hard and soft constraints

**DOI:** 10.1186/s13015-016-0070-z

**Published:** 2016-04-23

**Authors:** Ronny Lorenz, Ivo L. Hofacker, Peter F. Stadler

**Affiliations:** Institute for Theoretical Chemistry, University of Vienna, Währingerstrasse 17, 1090 Vienna, Austria; Research Group Bioinformatics and Computational Biology, Faculty of Computer Science, University of Vienna, Währinger Straße 17, 1090 Vienna, Austria; Center for non-coding RNA in Technology and Health, University of Copenhagen, Grønnegårdsvej 3, 1870 Frederiksberg, Denmark; Bioinformatics Group, Department of Computer Science, and Interdisciplinary Center for Bioinformatics, University Leipzig, Härtelstraße 16-18, 04107 Leipzig, Germany; Max Planck Institute for Mathematics in the Sciences, Inselstraße 22, 04103 Leipzig, Germany; Fraunhofer Institut for Cell Therapy and Immunology, Perlickstraße 1, 04103 Leipzig, Germany; Santa Fe Institute, 1399 Hyde Park Road, Santa Fe, NM87501 USA

**Keywords:** RNA folding, Dynamic programming, Constraints

## Abstract

**Background:**

A large class of RNA secondary structure prediction programs uses an elaborate energy model grounded in extensive thermodynamic measurements and exact dynamic programming algorithms. External experimental evidence can be in principle be incorporated by means of hard constraints that restrict the search space or by means of soft constraints that distort the energy model. In particular recent advances in coupling chemical and enzymatic probing with sequencing techniques but also comparative approaches provide an increasing amount of experimental data to be combined with secondary structure prediction.

**Results:**

Responding to the increasing needs for a versatile and user-friendly inclusion of external evidence into diverse flavors of RNA secondary structure prediction tools we implemented a generic layer of constraint handling into the ViennaRNA Package. It makes explicit use of the conceptual separation of the “folding grammar” defining the search space and the actual energy evaluation, which allows constraints to be interleaved in a natural way between recursion steps and evaluation of the standard energy function.

**Conclusions:**

The extension of the ViennaRNA Package provides a generic way to include diverse types of constraints into RNA folding algorithms. The computational overhead incurred is negligible in practice. A wide variety of application scenarios can be accommodated by the new framework, including the incorporation of structure probing data, non-standard base pairs and chemical modifications, as well as structure-dependent ligand binding.

**Electronic supplementary material:**

The online version of this article (doi:10.1186/s13015-016-0070-z) contains supplementary material, which is available to authorized users.

## Background

Despite its pervasive success in a wide variety of applications, thermodynamics-based pseudo-knot free RNA secondary structure prediction is by no means perfect [1, 2]. The same is true of SCFG-based approaches [3, 4]. It is therefore of key interest to guide the RNA secondary structure predictions by incorporating experimental evidence beyond the parameters of the standard energy model [5]. This view was emphasized already in [6] by proposing a constraint programming framework for RNA folding.

Thermodynamic folding software (mfold [7], ViennaRNA Package [8]) includes the possibility to constrain the set of allowed base pairs or to force individual nucleotides to be paired. Early approaches towards incorporating additional information, e.g. from chemical or enzymatic probing data or known chemical modification, used large energy penalties to effectively prohibit certain conformations [7]. We refer to such all-or-none decisions as *hard constraints*. A special case is the set of suboptimal structures sensu Zuker, which consists of the most stable secondary structures in which a single base pair is enforced as a hard constraint. All $O(n^2)$ Zuker-suboptimal structures can be computed simultaneously with specialized recursion in $O(n^4)$ time [9]. A very general framework for hard constraints are *thermodynamic matchers* [10]. Here, a kind of scaffold consisting of building blocks such as stems and loops with variable sizes can be specified. This specification is then translated into a specialized dynamic programming algorithm that computes the optimal folds or partition functions from the ensemble of structures that fit the prescribed scaffold. A convenient implementation is LocoMotif [11], which is built on top of algebraic dynamic programming (ADP) [12]. Abstract shapes [13] are a conceptually very similar way of specifying such scaffolds at different resolutions with the advantage that classified dynamic programming can be used to compute them.

Constraints are of key interest also in scenarios where RNAs interact with other RNAs, proteins, or small ligands. Hard constraints have been used to model the exposition of binding sites for RNA–RNA interactions [14–16], for RNA-protein interactions [17], and to constrain aptamer structures with bound ligands in the context of riboswitch design [18].

Instead of enforcing hard constraints, most of the more recent approaches use moderate “bonus energies” to reward (or penalize) structures that match (or contradict) external information. We refer to such additional pseudo-energy terms as *soft constraints*. The bonus energies are usually chosen proportional to some measure of signal strength or confidence. This idea is used e.g. in RNAalifold in the context of consensus structure prediction from aligned RNA sequences. Here sequence covariation supporting base pairs is rewarded by a small additional stabilizing pseudo-energy [19, 20]. A similar approach has been pursued more recently in TurboFold [21].

Soft constraints have gained considerable interest in the analysis of chemical and enzymatic probing experiments. In RNAstructure [22–24], SHAPE reactivities are converted to position-specific destabilizing energies for base pair stacks. The incorporation of SHAPE data into secondary structure prediction as soft constraints has been shown consistently to improve the accuracy of structure prediction [25]. The same idea, albeit with different models of bonus energies, has been used successfully also for other types of chemical probing e.g. using DMS [26] and to enzymatic probing (PARS [27, 28]). A variation on this theme has been proposed in [29]: instead of computing the bonus energies directly from the reactivities, they are determined so that the sum of bonus energies and deviations between predicted and measured signal together is minimized.

The increasing amount of experimental data that inform on secondary structures demands for RNA folding algorithms that can incorporate external information in a more principled and versatile manner. Increasing interest in chemically modified nucleotides, which play a particular role e.g. in tRNAs [30], on the other hand, suggests that hard constraints, in particular the exclusion of particular positions from pairing, are becoming features of practical interest.

The use of large energy penalties as a substitute for hard constraints entail several practical disadvantages. In particular, it drastically complicates the numerics of partition function calculations. Practical implementations of McCaskill’s algorithm e.g. in the ViennaRNA Package therefore avoided energy penalties and instead modeled hard constraints by ignoring certain cases in the dynamic programming recursions themselves.

Here we report on the comprehensive implementation of a broad array of both hard and soft constraints in the RNA folding algorithms of the ViennaRNA Package [8, 31].

## Hard constraints

### Consistency of constraints and recursions

Let us first consider the simplest model of RNA folding, the circular matching problem [32]. Its solution is based on the simple observation that each base pair (*k*, *l*) separates the RNA structure in an independent structure inside the base pair and an structure outside the pair. The different possible structures thus can be enumerated by recursively decomposing a structure on the sequence interval [*i*, *j*] as follows1One can therefore count the structures by means of the simple recursion

2 $$\begin{aligned} N_{ij} = N_{i+1,j} + \sum _{k=i+1}^n N_{i+1,k-1} N_{k+1,j} \end{aligned}$$

with the initialization $N_{ii}=1$ for all *i* and $N_{i,i-1}=1$. This version does not explicitly enforce the base pairing rules. Write $(i,j)\in \mathcal {B}(x)$ for the set of all pairs of positions that can form a base pair given the input string *x*. We include the base pairing constraint into Eq. (2) by simply constraining the sum:

3 $$\begin{aligned} N_{ij} = N_{i+1,j} + \sum \limits _{\begin{array}{c} k=i+1\\ (i,k)\in \mathcal {B}(x) \end{array}}^n N_{i+1,k-1} N_{k+1,j} \end{aligned}$$

Of course, this kind of base pairing constraint has been used explicitly in RNA folding algorithm. It can be seen, however, as a particularly simple example of a much larger class.

The most general constraints that do not require a fundamental change in the algorithm are those that cause the skipping of a particular term in the recursion (2). Clearly, these can be encoded by specifying the bases that can be unpaired (1st term) and the base pairs that may be formed [as in Eq. (3)]. Since bases cannot pair with themselves, it is convenient to use the diagonal of a constraint matrix $\mathbb {X}$ to address the unpaired nucleotides, i.e., we use $\mathbb {X}_{ii}=1$ if position *ican* be unpaired and $\mathbb {X}_{ii}=0$ if position *imust not* be unpaired. Correspondingly, $\mathbb {X}_{ij}=1$ means that position *i* is allowed to pair with position *j*, while $\mathbb {X}_{ij}=0$ implies that position *i* must not pair with position *j*. With this notation, recursion (2) is modified to

4 $$\begin{aligned} N_{ij} = \mathbb {X}_{ii} N_{i+1,j} + \sum _{k=i}^j \mathbb {X}_{ij}N_{i+1,j+1}N_{k+1,j} \end{aligned}$$

with the initialization becomes $N_{ii}=\mathbb {X}_{ii}\cdot 1$ for all *i*. We use a special typeface of the symbol $\mathbb {X}$ to emphasize that it is not implemented as multiplication by 1 or 0 but including or excluding the entire term from the computation. This saves the time for retrieving the stored values of *N* and computing the corresponding expression altogether.

The framework of ADP [12] makes it transparent that the structure of recursion is determined by the grammar that is used to generate the search space. This structure is also exposed explicitly when stochastic context-free grammars (SCFGs) [3] are used to formulate the RNA folding problem. The constraints can refer individually to each of the production rules. Furthermore, they are tied to the terminal symbols since only the terminals provide a direct link to sequence positions and energy values. We use here a diagrammatic version inspired by Feynman diagrams and Ref. [33] to specify the grammars that seems easier to interpret and allows us also to indicate the indices of the corresponding memo-tables. The classical recursions of the standard model of RNA folding have the following form:5Here *F* refers to arbitrary secondary structures, *C* represents structured enclosed by a base pair, *M* is an arbitrary component inside a multibranch loop, and $M^1$ is right-most component of a multibranch loop delimited by a base pair on its left end. We refer to the literature on RNA folding algorithms, in particular [31] and the references therein, for a detailed discussion.

This standard model can easily be augmented by attaching a binary constraint variable $\mathbb {X}^{\tau }_{\dots }$ to each alternative or symbol on the r.h.s. of the production. Here $\tau$ now denotes the different types of terminals, i.e., hairpin loops, interior loops, as well as the components of the multibranch loops. In particular, it is now possible to distinguish base pairs in different contexts: for instance, we could treat the right-most pair in a multibranch loop differently from all other interior delimiting base pairs in a multibranch loop by specifically constraining the *C*-term in the $M^1$ recursion but not the *C*-terms in *M* recursions. The standard recursions also are amenable to restricting the degree of multibranch loops to 2. This is easily achieved by forbidding the 2nd term in the *M*-recursion, enforcing that *M* also contains only a single component.

In practice it appears most interesting to put constraints on base pairs and unpaired positions and to make these constraints dependent on (a) the loop type, and (b) on whether the base pair is the closing pair or an interior pair of a multibranch loop or interior loop:

1. Unpaired bases in external sequence are treated in the *F*-recursion by constraining the first term;
2. Unpaired bases in hairpin or interior loops are dealt with in the first two terms of the *C*-recursion;
3. Unpaired bases in a multibranch loop context are treated in both the *M* and the $M^1$ recursion;
4. Closing base pairs for hairpin-, interior-, and multibranch loops are handled in the 1st, 2nd, and 3rd term of the *C*-recursion, respectively;
5. Interior base pairs for interior loops are treated in the 2nd term of the *C*-recursion, considering the (*k*, *l*) instead of the (*i*, *j*) pair. In this term one could also prohibit certain bulges or base pair stacking;
6. Interior base pairs in a multibranch loop context can be excluded by constraining the two terms in the *M*-recursion and the term in the $M^1$-recursion that contains *C*.

It is important to note that not all conceivable multibranch loop constraints can be implemented in a straightforward manner due to the special form of the linear multibranch loop decomposition. While we can directly prohibit the closing pair and each of the interior base pair separately, a constraint excluding only a particular combination of closing and interior base pair, i.e., a particular multibranch loop is not compatible with the grammar because the combination of the base pairs does not appear together in a single derivation. On the other hand, individual multibranch loops can be addressed and either forbidden or enforced. It is also impossible to formulate constraints such as “one of the base pairs (*i*, *j*) and (*k*, *l*) is formed” or “one of the positions *i* and *j* remains unpaired” in a manner that is consistent with grammar. The reason is, of course, that while the recursion operates on a particular base pair or unpaired base, it does not have any information on the other pair.

Hard constraints are commonly used in RNA folding. First, $\mathbb {X}_{ij}=0$ whenever sequence positions *i* and *j* do not satisfy the base pairing rules, i.e., $x_ix_j\notin \{GC,CG,AU,UA,GU,UG\}$ in the default setup. Second, $\mathbb {X}_{ij}=0$ for $|j-i|\le 3$ to enforce the geometric constraint on hairpin loops, which must consist of at least three unpaired nucleotides. The latter constraint, however, is usually implemented by modifying the sum from $\sum _{k=i}$ to $\sum _{k=i+4}$ rather than as an explicit constraint.

#### Limitations and relationship with classified DP

Not all useful combinations of constraints can be expressed in the current implementation. It provides access to base pairs, unpaired bases, loops, and the components of the multibranch loop decomposition as its literals. Literals and their negations can be combined only as conjunctions, i.e., by means of the logical AND operator. This is a severe restriction of the expressivity of the constraint language compared to the full power boolean functions on the constraints.

This limitation is, however, not a matter of an incomplete implementation. Conjunctions of constraints instead are essentially all that can be achieved without a memory of past decisions on which constraints to include. Although useful in practice, in constraints of the form “*S* contains at least one of the base pairs (*i*, *j*) and (*k*, *l*)” and “*S* does not contain (*k*, *l*) if *S* contains (*i*, *j*) but otherwise may contain (*k*, *l*)” cannot be expressed in the current framework because such constraints require a memory of past choices. In order to decide whether the base pair (*k*, *l*) can be, must be, or must not be inserted, we need to know whether (*i*, *j*) has been inserted or not. Since the decision on (*k*, *l*) depends on the status of (*i*, *j*), the algorithm has to branch out into both cases. Conditional constraints therefore can be handled only by means of *classified dynamic programming* similar to thermodynamic matchers [11] or RNAshapes [34]. The basic principle of classified DP is that each non-terminal represents an array, with each entry referring to a particular “class”, e.g., structures under the *condition* that they satisfy certain patterns of constraints that appear as preconditions for other constraints. This necessarily introduces a multiplicative overhead in both running time and memory that grows with the number of required classes. The framework provided here is therefore strictly limited to unconditional local choices.

### Constraints on base pairing span

Long range base-pairs are often considered less reliable, see e.g. [1, 35]. As a consequence, some studies are restricted to local base pairs. This constraint is easily implemented by setting $\mathbb {X}_{ij}=0$ for $|j-i|+1\le L$, for some fixed upper bound *L* of the base pair span. This does not, however, reduce the memory consumption of the normal folding algorithms (although it can provide a substantial speed-up).

Specialized memory efficient algorithms are available in the ViennaRNA Package that restrict the memory to windows of a length *w* with $L\le w\ll n$. These tools, are *not* strictly equivalent to constraining base pair span. RNAplfold [36] computes averages over base pairing probabilities over all possible windows of length *w* that contain the pair. RNALfold [37] provides local fragments of the minimum energy structure. We also plan to provide access to additional hard and soft constraints in these tools. Since these programs are geared towards use with very large RNAs, even genome-wide studies, a convenient interface will be provided only for position-wise soft constraints, although the full constraint handling machinery will be implemented and can then be accessed at the level of the RNAlib C-library, presumably starting with version 2.4 of the ViennaRNA Package.

### Efficient construction of the constraint matrix

The expression of constraints in terms of $\mathbb {X}^{\tau }_{ij}$ is both the most general and the algorithmically most natural representation. It is straightforward to exclude unwanted base pairs (*i*, *j*) or an unwanted unpaired nucleotide *i* in this way. It suffices to simply set $\mathbb {X}^{\tau }_{ij}=0$ and $\mathbb {X}^{\tau }_{ii}=0$, respectively. On the other hand, it is quite inconvenient to directly input the constraints required for enforcing base pairs or unpaired bases in this way.

In order to force a base *i* to remain unpaired it suffices to exclude all base pairs that involve *i*, i.e., $\mathbb {X}_{ki}=0$ for all $k<i$ and $\mathbb {X}_{ik}=0$ for all $k>i$. Similarly, we can enforce the base pair (*i*, *j*) by setting $\mathbb {X}_{ii}=\mathbb {X}_{jj}=0$, and forbidding all alternative pairing partners of *i* and *j*. Furthermore it makes sense to exclude all base pairs that are inconsistent with *i* and *j* since these cannot be included as well. Forcing a position *i* to be paired with an unspecified partner is achieved by simply setting $\mathbb {X}_{ii}=0$. If the partner is located upstream, we set $\mathbb {X}_{ij}=0$ for all $j\ge i$. These cases cover all constraints that are typically considered in RNA folding programs.

Enforcing an interior loop may be of interest for instance when certain 3D elements such as kink-turns are known *a priori*. In order to include the interior loop with the delimiting base pairs $(u',u'')$ and $(v',v'')$ with $u'<v'<v''<u''$ one has to (1) exclude all base pairs that are incompatible with $(u',u'')$ and $(v',v'')$, (2) exclude all terms in the *C*-recursion for $i=u'$ and $j=u''$ except the single term with $v'=k$ and $v''=l$, (3) enforce all nucleotides *w* with $u'<w<v'$ and $v''<w<u''$ to remain unpaired, and (4) one has to enforce the delimiting base pairs themselves by forbidding $u'$, $u''$, $v'$, and $v''$ to remain unpaired.

In principle there are $O(n^4)$ interior loops. This makes it impractical to list all possible constraints on them explicitly. As we have seen above, enforcing particular interior loops can easily be achieved by constraining both of their delimiting base pairs and forcing the remaining bases of the loop to remain unpaired. Since more than *n*/2 base pairs (and hence also the loops they close) are necessarily in inconsistent, any list of enforced interior loops can be stored efficiently. Prohibiting interior loops is also easy from the algorithmic point of view: It suffices to skip a particular combination of closing pair and interior delimiting pair. To avoid the storage of an $O(n^4)$ constraint matrix, forbidden interior loops are stored in a sparse data structure as described below in the section on generalized constraints.

A key observation is that a given set of constraints often implies additional constraints that ideally should be used in the folding recursions. In [6] a constraint satisfaction framework is used that nominally requires $O(n^4)$ time to propagate the constraints. We remark here that the constraint matrices $\mathbb {X}$ for base-pair level constraints can be computed in cubic time, assuming that the list of input constraints is non-redundant. Although loop-type-dependent constraints can be handled analogously (and are supported in the implementation) we describe the constraint propagation algorithm here only for context-independent constraints on base pairs:

First we observe that there are no more than $n(n+1)/2$ base pairs or unpaired bases to exclude. Each of these constraints can be handled in constant time by setting a single $\mathbb {X}_{ij}$ entry to 0. In order ensure that a nucleotide, say *i*, remains unpaired we have to set the entries for its $n-1$ possible pairing partners to 0. The total effort for unpaired positions thus is $O(n^2)$. Since a secondary structure cannot contain *n*/2 or more base pairs, the constraints cannot specify more than *n*/2 enforced base pairs. Thus we first check the list of enforced pairs for consistency. This can be done in at most quadratic time. For every pair (*p*, *q*) in the list, we have to set at most $O(n^2)$ entries to 0, hence the total effort is not more than $O(n^3)$.

An important issue is that multiple constraints may be inconsistent. This is of course easily checked for enforced base pairs. A necessary condition that can be checked efficiently after inserting a constraint is that each nucleotide can be either unpaired or paired with at least one partner, i.e., $\sum _{j=1}^{i} \mathbb {X}_{ji} + \sum _{j=i+1}^{n} \mathbb {X}_{ij}>0$ for all *i*. This condition is not sufficient, however.

## Soft constraints

### Unpairing and pairing penalties

In contrast to hard constraints, which restrict the search space, the inclusion of soft constraints only biases the ensemble of secondary structures to either favor or discourage certain structures or structural features. The search space remains unchanged. Soft constraints are naturally implemented in folding algorithms using bonus energies. Similar to hard constraints, a versatile set of soft constraints can be incorporated into folding algorithms without changing the underlying structure of the recursions.

The most prominent type of soft constraint refers to whether a particular nucleotide position *i* is paired or unpaired. More precisely, we add to the energy of a particular secondary structure $\psi$ of a sequence *x* a bonus energy $b^p_i$ if we have a certain amount of evidence to position *i* is paired and an energy contribution $b^u_i$ if *i* is unpaired in $\psi$. Note that whether or not *i* is unpaired is determined by the secondary structure $\psi$. The modified energy $E(\psi )$ can be written as

6 $$\begin{aligned} E(\psi )\,= & {}\, E_0(\psi ) + \sum _{i\in \psi ^p} b^p_i + \sum _{i\in \psi ^u} b^u_i \\ = &\, E_0(\psi ) + \sum _{i=1}^n b^p_i + \sum _{i\in \psi ^u} (b^u_i-b^p_i) \nonumber \\= & {}\, E_0(\psi ) + E' + \sum _{i\in \psi ^u} \delta _i \end{aligned}$$

where $E_0(\psi )$ is the energy of $\psi$ as evaluated in the standard energy model, $E'$ is a constant independent of the $\psi$ and $\delta _i := b^u_i-b^p_i$ is a single position-dependent bonus energy parameter. Since a shift $E'$ in the energy scale of all structures does not affect the ensemble of secondary structures, Eq. (6) shows that a single position-dependent parameter added *only* to the unpaired bases is sufficient to describe all position-dependent effects on the ensemble of secondary structures.

Base pairs can be treated analogously. Conceptually, it may be of interest to not only reward the formation of a particular base pair but also to discourage a particular pair without forcing either one of the involved bases to be unpaired. Denote by $b^p_{ij}$ a bonus terms added to energy if the the base pair (*i*, *j*) is formed, and let $b^u_{ij}$ be a penalty for not forming the base pair (*i*, *j*). A short calculation analogous to Eq. (6) shows that is is again sufficient to attached a contribution to the base pairs:

7 $$\begin{aligned} E(\psi )\,= &\, {} E_0(\psi ) + \sum _{(i,j) \in \psi } b^p_{ij} + \sum _{(i,j) \notin \psi } b^u_{ij} \\ =& \, E_0(\psi ) + \sum _{i<j} b^u_{ij} + \sum _{(i,j) \in \psi } (b^p_{ij}-b^u_{ij}) \nonumber \\= &\, {} E_0(\psi ) + E' + \sum _{(i,j) \in \psi } \Delta _{ij} \end{aligned}$$

where $\Delta _{ij}:= b^p_{ij}-b^u_{ij}$ is again the difference of the original bonus energies. Hence, it suffices to keep track of base pair specific soft constraints within a single upper triangular matrix $\Delta$.

From the algorithmic point of view it is straightforward to incorporate this type of conditional soft constraint into the energy evaluation of the *C*-terms, i.e., to add $\Delta _{ij}$ to the energy of the closing pair of each loop.

### Constraining base pairing span

A recent publication [38] proposed to reduce the occurrence of long-range base pairs by down-weighting the energy of long-range base pairs. To this end, the free energy contribution $E^L_{ij}$ of a loop *L* closed by the base pair (*i*, *j*) is modified by a factor

8 $$\begin{aligned} \gamma (i,j) = \alpha _1 \cdot (e^{-\frac{j-i-1}{\alpha _2}} - 1) + 1 \end{aligned}$$

where $\alpha _1$ and $\alpha _2$ are control parameters that fine-tune the steepness of the decay function $\gamma$. The recursion then use $\tilde{E}^L_{ij} = \gamma (i,j) \cdot E^L_{ij}$ instead of $E^L_{ij}$. Since our ansatz of soft constraints is additive and not multiplicative, this down-weighting can not be expressed easily in the above framework. Nevertheless, it can be accomplished using the generic soft constraints feature described below, where one simply substracts $E^L_{ij}$ followed by adding $\gamma (i,j) \cdot E^L_{ij}$. It should be noted, that such multiplicative modifications lack a direct thermodynamic interpretation. As an alternative, the down-weighting of long-range base pairs can be relegated to a modified MEA computation based on the unaltered base pairing probability matrix [39].

#### Loop type dependent soft constraints

In complete analogy with the binary constraint variable $\mathbb {X}^\tau _{ij}$ used for hard constraints, a single, real-valued soft constraint variable $\Delta ^\tau _{ij}$ with $\Delta ^\tau _{ii} = \delta ^\tau _i$ containing position-wise bonus energies is used to specify the entire set of pseudo-energies for unpaired nucleotides and base pairs. Again, it is straightforward to use different values for different types $\tau$ of loops. The total free energies $\tilde{E}^\tau _{i,j}$ of any loop $\tau$ with closing pair (*i*, *j*) can now be computed by

9 $$\begin{aligned} \tilde{E}^\tau _{ij}&= E^\tau _{ij} + \Delta ^\tau _{ij} + \sum _{u \in \tau } \Delta ^\tau _{uu} \end{aligned}$$

where $E^\tau _{ij}$ is the energy contribution of $\tau$ as specified by the nearest neighbor energy model, and *u* denotes unpaired nucleotides within the loop.

*Generalized soft constraints* A simple extension of $\Delta$ to context aware pseudo-energies $\Delta ^\tau$ cannot readily cover all possible cases that appear as a single decomposition step of the RNA folding grammar. Interior loops, for instance are evaluated as a single component, where both, the closing pair (*i*, *j*) and the enclosed base pair (*k*, *l*) are evaluated at the same time. However, $\Delta ^\tau$ does not allow for conditional contributions of the form: (*k*, *l*) delimits an interior loop closed by (*i*, *j*), and vice versa.

The most natural way to deal with this restriction is to make the soft constraint explicitly dependent on each of the decomposition steps $d\in \mathbb {D}$ that appear in the folding grammar, together with their corresponding delimiting nucleotide positions. The grammar of the standard nearest neighbor energy model, Eq. (5), yields 10 different cases with at most four delimiting nucleotide positions, hence $\Delta$ requires an extension to $\Delta _{ij(k)(l)}^d$. As a consequence, storing all possible soft constraints in additional triangular matrices requires a memory overhead of $\mathcal {O}(n^4)$. In practice, however, such constraints are typically derived from rules that e.g. put penalties on particular sequence motives, or structural features, for instance the loop’s asymmetry, that can be efficiently computed on the fly. In our implementation, we therefore represent such complex constraints as a function

10 $$\begin{aligned} f: \mathbb {N}^m \times \mathbb {D} \rightarrow \mathbb {R} \end{aligned}$$

that returns the bonus energy. The user is then free to provide essentially arbitrary bonus energy models that may combine rules and elaborate parametrizations. These are then defined and stored external to the core RNA folding algorithms.

### Interval constraints

Although hard constraints as defined above can be used to enforce unpaired intervals representing e.g. protein binding sites, this is not amenable to soft constraints that refer to entire sequence intervals as a unit.

Let us consider the following model. We are given a collection of *M* binding sites $[p_i,q_i]$, each associated with a bonus energy $\beta _{ij}$, e.g. from binding a certain protein. Each interval $[p_i,q_i]$ can be either occupied or not. A special case of this problem has in fact already been dealt with in the ViennaRNA Package, namely the incorporation of G-quadruplexes [40]. This amounts to extending the recursions of Eq. (5) by identifying intervals that are unavailable for secondary structure formation:11In principle, it suffices to just change the energy contribution.

It may be undesirable, however, to treat the binding sites, such as G-quadruplexes, as structural domain. Instead, one can explicitly treat them like unpaired stretches in their native loop context. This naturally leads to the following modified RNA folding grammar, which with some minor modifications has already been used in [17]:12The idea here is that motive based terms *B* are added to the hairpin, interior and multibranch loop contributions that appear explicitly as intervals in their recursions. If desired, this self-contained recursion can be used separately for the different loop types.

Of course, it is possible to combine this approach to include binding sites with the G-quadruplex grammar. Again, we consider here only the case where a single type of binding site contributions *B* is computed:13Binding site contributions may also be scored with different pseudo-energies in different loop contexts. In this case different variants of the last recursion, which computes the energies of unpaired stretches containing a pattern of binding sites, need to be scored differently for particular loop contexts.

### Hard constraints *versus* soft constraints

It is possible to emulate hard constraints by means of (large) soft constraints as in early implementations of mfold [41] and the ViennaRNA Package [8]. This has several disadvantages, however. Most importantly, large bonus energies cause numerical problems in the computation of partition functions. Stochastic backtracing furthermore sometimes returns forbidden structures as these are only highly discouraged but not completely excluded.

Conversely it is possible to construct soft constraints using multiple structure predictions with hard constraints. If a particular structural feature that can be expressed as a hard constraint $\mathbb {X}$ is associated with a pseudo-energy $E(\mathbb {X})$, then the modified MFE is $\min ( \text {MFE}, \text {MFE}[\mathbb {X}] + E(\mathbb {X}))$, where $\text {MFE}[\mathbb {X}]$ refers to the minimum free energy computed with the constraint $\mathbb {X}$. Analogously, the partition function of the distorted ensemble is

14 $$\begin{aligned} Z' = (Z-Z[\mathbb {X}]) + Z[\mathbb {X}] e^{-E(\mathbb {X})/RT} \end{aligned}$$

Derived quantities can be computed as linear combinations of the distorted and the undistorted ensemble. We consider the base pairing probabilities as an example. First we observe that base pairing probabilities in the original ensemble under the condition that constraint $\mathbb {X}$ is *not* satisfied can be computed from base pairing probabilities $p_{ij}$ is the unconstrained ensemble and $p_{ij}[\mathbb {X}]$ in the ensemble constrained by $\mathbb {X}$ with the help of the two partition functions *Z* and $Z[\mathbb {X}]$ as weighted difference

15 $$\begin{aligned} p_{ij}[\lnot \mathbb {X}] = p_{ij} - \frac{Z[\mathbb {X}]}{Z} p_{ij}[\mathbb {X}] \,. \end{aligned}$$

Since the soft constraint on $\mathbb {X}$ shifts all structures that satisfy $\mathbb {X}$ by the same Boltzmann factor $\exp (-E(\mathbb {X})/RT)$ we have

16 $$\begin{aligned} p'_{ij} = \frac{Z-Z[\mathbb {X}]}{Z} \cdot p_{ij}[\lnot \mathbb {X}] + \frac{Z[\mathbb {X}]}{Z} \cdot p_{ij}[\mathbb {X}] e^{-E(\mathbb {X})/RT} \end{aligned}$$

The difficulty with this approach, as noted e.g. in [17] is that the simultaneous inclusion of *K* soft constraints requires the computation of $2^K$ soft constraints. The reason is that the soft constraints are not independent and thus the joint effect of $\mathbb {X}_1$ and $\mathbb {X}_2$ has to be modeled using the inclusion/exclusion principle, i.e., “$(\mathbb {X}_1\cup \mathbb {X}_2) = \mathbb {X}_1 + \mathbb {X}_2 - (\mathbb {X}_1\cap \mathbb {X}_2)$” and so on. This approach thus quickly becomes inefficient because the number of terms increases exponentially with *K*. The equivalent soft constraints, on the other hand, require only a small multiplicative overhead caused by the need for a more elaborate energy evaluation associated with the individual steps in the recursion.

## Implementation issues

Both hard and soft constraints as defined in the previous sections are most naturally implemented as an additional layer interleaved between the structural decomposition, i.e. the productions of the grammar that generates the search space, and the energy evaluation of the standard nearest neighbor energy model.

Hard constraints $X^\tau _{ij}$ are efficiently stored as bit vectors in an upper triangular matrix, where any bit vector holds the entries of the $\tau$ dimension. The standard secondary structure model distinguishes only four types of loops, namely exterior, hairpin, interior, and multibranch loops. For each loop we distinguish between the closing pair and the enclosed pairs. The exterior loop has no closing pair, while hairpin loops have no enclosed pairs. Altogether, we therefore distinguish six types of base pairs: (1) enclosed in the exterior loop, (2) closing a hairpin loop, (3) closing an interior loop, (4) enclosed by an interior loop, (5) closing a multibranch loop, and (6) enclosed by a multibranch loop. Hard constraints therefore are conveniently stored in eight bit characters, requiring a total of $n(n+1)/2$ bytes of memory.

For each unpaired nucleotide *i* in a loop, a look-up in the corresponding constraint matrix $X_{ii}$ is required. Large loops therefore may create a substantial overhead slowing down the computation. The decomposition rules for secondary structures ensure that stretches of unpaired nucleotides in a loop are consecutive along the backbone. We therefore precompute an index storing for each sequence position the number of consecutive nucleotides that have to remain unpaired. This reduces the number of look-ups for nucleotide-wise constraints to just two for interior loops, and a single one in each derivation step for all the other loop types.

Soft constraints require as much memory as one of the triangular DP matrices because explicit energy values need to be stored. Thus a sparse matrix approach might be useful if only a small number of soft constraints is provided. We nevertheless use full matrices in the current implementation for the sake of computational speed. Furthermore, we precompute the pseudo-energy contributions of consecutive unpaired nucleotides starting at position *i* up to the end of the sequence in a second, auxiliary matrix. Similar to hard constraints, this speeds up the computations for larger loops since unpaired nucleotides induce only one or at most two additional arithmetic operations.

Therefore, usage of the soft constraint feature in the library or the interactive programs of the ViennaRNA Package incurs a memory overhead of 50 % compared to the folding algorithms without constraints.

The generalized constraint feature is implemented by pointers to user-defined callback functions. These functions are given a set of sequence coordinates and the type of decomposition to (1) evaluate the corresponding pseudo-energy contribution (soft constraints), or (2) evaluate whether a decomposition should be processed or skipped (hard constraints). An additional callback function may be specified to serve as a method to initialize any pre-, or post-conditions. These functions are called by our implemented prediction algorithms before, while, and after the actual dynamic programming recursions. Furthermore, the implementation provides an additional pointer that may be set to an auxiliary, arbitrary data structure that can be used e.g. to store precomputed data required for the pseudo-energy contribution callback.

The computational overhead of the generalized constraint features naturally depend strongly on the complexity of the callback functions. However, since a single pseudo-energy callback function is used to cover all decomposition steps, this function will be called $n^3$ times and spawn additional $n^3$ additions, or multiplications for MFE, or partition function computations, respectively. Hence, even for a callback function that implements a table look-up in $\mathcal {O}(1)$ a slight increase in computation time is expected.

For a detailed performance comparison see the Additional file 1.

*Input formats* As stated before, different subsets of the hard-, and soft-constraint paradigms outlined here have already been used by RNA structure prediction programs for several years. The programs of the ViennaRNA Package, for instance, have been using a simplified subset of loop-type unaware hard constraints that can be expressed as pseudo-dot-bracket notation consisting of the characters $< \,> \,. \,| \,( \,)\, {\times}$ (see the Additional file 1 for details of this format). The drawback of this format is that it represents at most one constraint for each sequence position. It is therefore not suitable for more complex hard constraints. Although we provide now a more sophisticated interface, this simple way of expressing hard constraints will be maintained for convenience and backward compatibility.

In addition, we introduce a generalization of the constraints file format used in UNAfold/mfold, to expose a larger subset of the new features through several executable programs shipped with the ViennaRNA Package, e.g. RNAfold, RNAalifold, and others. This basic set consists of loop-type dependent hard constraints for single nucleotides and base pairs, as well as simple soft constraints for unpaired nucleotides and base pairs. A detailed specification for these constraint definition files can be found in the Additional file 1.

The remaining functionality, e.g., the generalized constraints feature, is only accessible through the API of the ViennaRNA C-library, and through the scripting language interface.

For the sake of convenience, many programs of the ViennaRNA Package can parse text files that contain normalized SHAPE reactivity data (see Figure S2 in the Additional file 1). This data is then automatically converted into pseudo-energy contributions for the soft constraints feature [42]. Furthermore, we provide a reference implementation for the use of the generic soft constraints feature that allows for the inclusion of ligand binding to hairpin, and interior loop motifs. The feature is readily available through the C-library, the scripting language interface, and as an optional command-line parameter of the program RNAfold.

## Application scenarios

### Hard constraints

#### Speed-up for stochastic backtracing

**Fig. 1:**
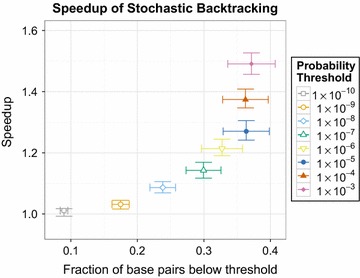
Speedup gained from removing low probability base pairs. Speedup for drawing 1,000,000 samples from the Boltzmann ensemble due to removal of low probability thresholds. The speedup largely depends on the fraction of base pairs the can be removed

Stochastic backtracing algorithms [43, 44] can be made more efficient by altogether omitting very rare base pairs. More precisely, if we need a sample of *N* structures of length *n* we expect not to see base pairs that appear with a pairing probability smaller than 1/(*nN*). As recently proposed by Aalberts in a talk at the Benasque 2015 meeting, sampling can be made more efficient by forbidding very rare base pairs completely. Figure 1 shows that the gain in the current implementation is rather moderate, reaching less than a factor of 2. Much larger values should be achievable by implementing e.g. the Boustrophedon method [45].

#### Towards more accurate tRNA structure prediction

Chemical modifications of RNA molecules have recently been found to be a surprisingly abundant and diverse phenomenon. So far, more than a hundred different types of chemical modifications have been described [46]. In particular, tRNAs have long been known to be heavily modified. Many of these modifications are known to effectively prevent nucleotides from pairing [47] and in some specific examples (such as tRNA-Lys in human mitochondria [48]) the chemical modifications are necessary to ensure that the RNA folds into its functional structure [49].

**Fig. 2:**
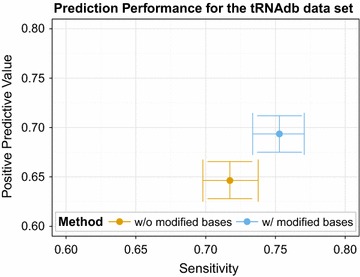
Prediction performance for tRNAdb benchmark set. Treating chemically modified bases as unpaired increases both sensitivity and positive predictive value on the tRNAdb benchmark set. 95 % confidence intervals were estimated using bootstrapping with 1000 iterations

To quantify the impact of this effect we retrieved all 606 tRNA sequences with known chemical modifications from the tRNAdb [50] and predicted secondary structures with and without constraints for non-pairing nucleotides. Figure 2 summarizes the expected improvement of the predicted structures when the modification constraints are taken into account.

### Soft constraints

#### Chemical probing methods

Chemical and enzymatic probing can provide information on the RNA structure at nucleotide resolution. The common theme of these methods is that RNA can be selectively modified or cleaved by small organic molecules, metal ions or RNAse enzymes. As adducts frequently lead to termination of reverse transcription, sequencing of the resulting cDNA fragments yields a position-wise signal of the processing propensity. We refer to [51] for a recent review. Incorporating knowledge on the typical structure-dependent patterns produced by a particular reagent, this signal can be incorporated as constraints into computational structure predictions algorithms. Most conveniently this is achieved by converting the measured signal into pseudo-energies that are associated with the paired and/or unpaired state of a given base. Starting with [22], heuristic conversions have been proposed and tested [23, 24, 27]. More recently, a more principled approach derived a pseudo-energy of the form $-RT\ln p_i/(1-p_i)$, where *p* is the probability that base *i* is unpaired given the probing measurement for base *i*. As described above, the ViennaRNA Package now provides a convenient interface to include arbitrary position-dependent bonus energies. The conversion of probing signals to pseudo-energies thus can be separated completely from the folding algorithms. We have already implemented and made available the most commonly used strategies for including SHAPE reactivity data in [42].

A recent, more detailed analysis of SHAPE probing data shows that the distribution of SHAPE signals is different for unpaired nucleotides, the interior pairs of stems, and the ends of stems [25]. This suggests to use a ternary model that applies different signal-dependent energy contributions for unpaired nucleotides, nucleotides involved in stems, and for base pairs delimiting stems. The loop decomposition of secondary structures gives us the following characterization:

A base pair (*i*, *j*) is the terminal pair of a stem if it is (a) the closing pair of a loop or if it (b) appears as the pair inside a loop that this not a stacked pair.

Unfortunately, these two cases are not disjoint in general. A base pair appears in both category (a) and category (b) if and only if it is an isolated “lonely” base pair. Thus this decomposition becomes unique when isolated pairs are excluded, which is possible in the ViennaRNA Package (using the --noLP option in the interactive programs).

As in Eq. (6) it suffices to attach bonus energies to unpaired nucleotides and to the terminal nucleotides of stems because

17 $$\begin{aligned} E(\psi )\, =\, E_0(\psi ) + E' + \sum _{i\in \psi ^t} \delta ^t_i + \sum _{i\in \psi ^u} \delta ^u_i \end{aligned}$$

where $E'=\sum _{i} b^s_i$, $\delta ^t_i = b^t_i-b^s_i$, $\delta ^u_i = b^u_i-b^s_i$, and $b^s_i$, $b^t_i$, and $b^u_i$, respectively are the position-wise bonus energies for nucleotides in the interior of stacks, at the stack ends, and unpaired nucleotides, respectively.

#### Assisted folding

The basic idea of “assisted folding” is to determine bonus energies not directly from measured data but to determine them from an error minimization problem [29, 52]. Both the standard energy model and the measured properties $\varphi _u$ of the secondary structure ensemble are considered to have errors. Suppose that a vector of the measured properties $\mathbf {\psi }(\mathbf {\varepsilon })$ can be predicted with a folding algorithm using the standard energy model and a vector of correction energies $\mathbf {\varepsilon }$. Then the most general form of the assisted folding problem becomes the joint estimation of the structural ensemble and the perturbation energies as an error minimization problem:

18 $$\begin{aligned} \mathbb {E}(\mathbf {\varphi },\mathbf {\varepsilon }) = \Vert \mathbf {\psi }(\mathbf {\varepsilon }) - \mathbf {\varphi }\Vert | + \Vert \mathbf {\varepsilon }\Vert \rightarrow \min \end{aligned}$$

where $\Vert \,.\,\Vert$ denotes a suitable norm. A special case of this approach has been exploited in [29], using position specific bonus energies $\varepsilon _i$ and the usual, variance-weighted euclidean norm. The same interface could in principle be utilized to implement a full-fledged Bayesian approach [52] incorporating essentially arbitrary models relating position-wise secondary structure to probability distributions of probing readout.

#### RNA-ligand interactions

Bacterial genomes have been found to harbor a large variety of metabolite sensing riboswitches [53]. By binding a metabolite ligand to specific sequence and/or structure motifs of the RNA, gene transcription and expression is regulated to allow adaptation to certain environmental needs. Upon sufficiently large concentrations of the ligand, the RNA “switches” into a corresponding structural state, stabilized by non-covalent binding, such that the RNAs native conformation is hardly adopted, hence the name riboswitch. The mode of control can either be positive or negative, i.e., activating or repressing. Furthermore, riboswitches in prokaryotes may function both transcriptionally and translationally. In eukaryotes, on the other hand, metabolite sensing is often indirect via proteins that then bind RNA and appears to be restricted to post-transcriptional regulation [54].

To model RNA-ligand binding to a single binding motif $\mathbb {X}$, existing methods usually make use of hard constraints, together with the experimentally determined dissociation constant of the RNA-ligand interaction. Analogously to Eq. (14), the partition function for all states, including those with bound ligand, is

19 $$\begin{aligned} Z' = Z + Z[\mathbb {X}]e^{-\Delta G/RT}. \end{aligned}$$

Thus, it can be easily constructed from the unconstrained partition function *Z*, and the partition function of those structures that exhibit the motif and bind the ligand $Z[\mathbb {X}]$. To account for the stabilization of the bound ligand, the concentration dependent binding free energy

20 $$\begin{aligned} \Delta G = RT \ln \frac{K_d}{c} \end{aligned}$$

is used.

**Fig. 3:**
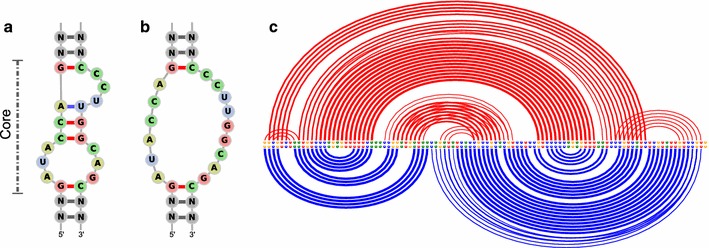
Theophylline ligand binding to RNA structure motif using the soft constraints framework.** a** The core motif of the aptamer conformation.** b** Core motif abstraction to a simple interior loop suitable for the soft constraints framework.** c** Shift in predicted equilibrium base pair probabilities from ligand free prediction (*upper arcs*), to prediction with bound theophylline (*lower arcs*) for an artificial RNA sequence taken from [58]

However, as mentioned above, this approach is not applicable to situations where an RNA has multiple binding sites. The implementation of generalized soft constraints, on the other hand, allows for a direct inclusion of $\Delta G$ to the binding motif, if it can be expressed as a hairpin or interior loop. For instance, the well studied theophylline aptamer is a relatively small interior loop motif with high sequence specificity that has a strong binding of $K_d = 0.32 \, \mu M$ [55], see Fig. 3. This aptamer has previously been applied in many designs of artificial RNA switches and different expression platforms [18, 56]. However, to our knowledge, the stabilizing effect of the theophylline binding has never been directly incorporated into the secondary structure design algorithms (other than using hard constraints).

As a soft constraint, the ligand binding can be modeled by simply adding the binding free energy $E_s = -9.22$ kcal/mol ($c=1\mathrm {mol}/\mathrm {L}$, $T=37$ °C) together with a small correction for the internal helix of three base pairs, whenever the interior loop (Fig. 3b) is encountered by the DP recursions. As a consequence, the shift towards the functional ligand binding state of the RNA under presence of theophylline is clearly visible in the base pair probabilities, see Fig. 3c.

To demonstrate the power of generic soft constraints, we have added a simple reference implementation for ligand binding in hairpin, and interior loops to the RNAlib C-library of the ViennaRNA Package. Potential motifs on the RNA are scanned in a preprocessing step, to create a list of target positions, which is then used for fast retrieval of the pseudo-energies in the MFE and partition function recursions for single RNA sequences. To provide a convenient interface we added the ligand motif feature as a command-line parameter to the RNAfold program. It can also be accessed easily from the scripting language interfaces. We refer to the RNAfold manpage and the reference manual of the RNAlib for further details.

## Concluding remarks

Most RNA folding program implement an ad hoc subset of the constraints for specific applications. Here we have presented a systematic way to include both hard and soft constraints into RNA folding programs. Both hard and soft constraints are implemented in the ViennaRNA Package starting from version 2.2 as an extra layer interleaved between the individual steps of the recursions and evaluation of the energy model. Hard constraints simply prune the alternatives in the recursions, while soft constraints add “bonus energies” to the standard energy model. Although hard and soft constraints can in principle be converted into each other, this is usually not efficient from a computational point of view. Therefore both types of constraints are handled simultaneously.

In order to accommodate the increasing interest in modeling protein binding to RNAs we have modified the grammar underlying the folding recursion to include unpaired intervals as elementary objects, similar to [17] and the handling of G-quadruplex structures [40]. These extensions, available with version 2.3 of the ViennaRNA Package, are only used when interval-type soft constraints are specified in the input and hence cause a computational overhead only when absolutely necessary. The generic layer of constraint handling is designed to be extensible. This facilitates in particular the implementation of future variants of “assisted folding” schemes without the need to touch the core folding routines.

As a proof of concept we recently used the new constraint handling facilities to provide interactive tools for incorporating SHAPE sequencing data into the ViennaRNA Package by means of three already existing strategies [42]. Here we briefly touch upon a variety of different application scenarios to demonstrate the versatility of the current implementation and interface. As we have seen, the consistent inclusion of hard and soft constraints makes it quite easy to address a wide array of specific issues that previously required non-trivial programming efforts to modify the folding programs themselves.

## Additional file

10.1186/s13015-016-0070-z Speedup gained from removing low probability base pairs (full data). **Figure S2:** Input file formats for the new hard/soft constraint features of the ViennaRNA Package. **Figure S3:** The computational overhead of the additional layers that facilitate hard/soft constraint support. **Table S1:** Full performance data for tRNAdb benchmark set.
