# Supplementary material for: RNA folding with hard and soft constraints
Source: Algorithms Mol Biol. 2016 Apr 23;11:8. doi: 10.1186/s13015-016-0070-z (PMC4842303; doi:10.1186/s13015-016-0070-z)
Supplement: Supplementary file 1 — 10.1186/s13015-016-0070-z Speedup gained from removing low probability base pairs (full data). Figure S2: Input file formats for the new hard/soft constraint features of the ViennaRNA Package. Figure S3: The computational overhead of the additional layers that facilitate hard/soft constraint support. Table S1: Full performance data for tRNAdb benchmark set. [file 13015_2016_70_MOESM1_ESM.pdf]

RNA Folding with Hard and Soft Constraints - Supplementary Material

Ronny Lorenz [1,*], Ivo L. Hofacker [1,2,3], and Peter F. Stadler [4,1,3,5,6,7]

[1]Institute for Theoretical Chemistry, University of Vienna, Währingerstraße 17/3, A-1090 Vienna, Austria.
[2]Research Group Bioinformatics and Computational Biology, Faculty of Computer Science, Univ. Vienna, Währingerstraße 17/3, A-1090 Vienna, Austria.
[3]Center for non-coding RNA in Technology and Health, University of Copenhagen, Grønnegårdsvej 3, 1870 Frederiksberg, Denmark.
[4]Bioinformatics Group, Dept. of Computer Science, and Interdisciplinary Center for Bioinformatics, University Leipzig, Härtelstraße 16-18, D-04107 Leipzig, Germany.
[5]Max Planck Institute for Mathematics in the Sciences, Inselstraße 22, D-04103 Leipzig, Germany.
[6]Fraunhofer Institut for Cell Therapy and Immunology, Perlickstraße 1, D-04103 Leipzig, Germany.
[7]Santa Fe Institute, 1399 Hyde Park Rd., NM87501 Santa Fe, USA.

1 Application Scenarios - Analysis Data

This section contains the full analysis data for which only concluding aspects are shown in the main manuscript.

1.1 Speed-up for stochastic backtracing

Speed-up gained from removing low probability base pairs was measured for different RNA sequence lengths and various base pair probability thresholds. See Figure S1 for details.

1.2 Towards more accurate tRNA structure prediction

Chemical modifications that are known to prevent nucleotides from pairing were used as hard constraints for the MFE structure prediction of tRNAs taken from `tRNAdb` [2]. In the main manuscript, we show the averaged prediction performances with and without application of hard constraints, while the detailed benchmark results for each data set are available in Table S1.

*To whom correspondence should be addressed

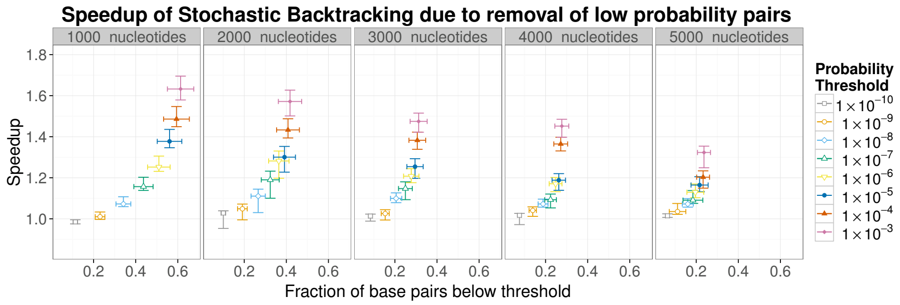

Figure S1: **Speedup gained from removing low probability base pairs (full data)**.

Stochastic backtracking speedup due to removal of low probability base pairs. $1,000,000$ samples where drawn from the Boltzmann ensemble using five sets of randomly generated RNA sequences with a length of $1,000$, $2,000$, $3,000$, $4,000$, and $5,000$ nucleotides, respectively. For each sequence length 16 sequences were generated to obtain an average value for the fraction of base pairs below a certain threshold, and the corresponding speedup upon removal of such pairs from the secondary structure space using hard constraints.

2 Input file formats for constraint handling

Constraint definition file

The programs of the `ViennaRNA Package` provide an easy to use interface to specify the new hard and soft constraints. For that purpose, we constructed a plain text input file format, where each constraint is given as a line of white-space delimited commands. The syntax we use generalizes the one used in `mfold`/`UNAfold` [3] for hard constraints, where each line starts with a command character followed by a set of positions. Our generalization of this format, however, introduces an extension of the command set to account for soft constraints, and some special cases that would otherwise require a larger set of multiple commands. Furthermore, each constraint command line may optionally be appended by a sequence of characters that identify a certain loop type context, as well as an orientation flag that enables one to force a nucleotide to pair upstream, or downstream. The full set of valid commands is listed in Figure S2B.

SHAPE reactivity file

Since several programs already implement a convenience command-line parameter switch to read and incorporate SHAPE reactivity data, a file format for position-wise normalized SHAPE reactivities is required as well. Since we intended to avoid re-inventing the wheel, the programs of the `ViennaRNA Package` happily accept the same input format as required for `Fold` of the `RNAStructure`

Dataset	Size	Nucleotides			Performance					
					w/o mod.			w/ mod.		
		total	mod.	block	PPV	TPR	cloverleaf	PPV	TPR	cloverleaf
Bacteria	139	10936	869	66	0.663	0.766	83/139	0.687	0.783	86/139
Archaea	76	5924	459	59	0.685	0.799	44/76	0.687	0.786	41/76
Eukaryotes (1)	242	18841	2982	574	0.604	0.685	128/242	0.684	0.753	144/242
Eukaryotes (2)	111	7993	720	125	0.605	0.661	47/111	0.646	0.687	44/111
Eukaryotes (3)	38	2972	307	17	0.694	0.768	22/38	0.729	0.796	23/38
Eukaryotes (4)	391	29806	4009	716	0.613	0.687	197/391	0.678	0.739	211/391
tRNAdb (total)	606	46666	5337	841	0.635	0.719	324/606	0.681	0.755	338/606

Table S1: **Full performance data for `tRNAdb` benchmark set.**
Prediction performances of MFE structure predictions for tRNAs with and without marking of modified (mod.) bases in terms of base pair formation inhibiting (block) hard constraints. For completeness, the proportion of predicted structures that shows a cloverleaf conformation is also listed. For Eukaryotes we show benchmark results for (1) nuclear, (2) mitochondrial, (3) plastid, and (4) all tRNAs.

package[1]. Thus, reactivity data must be stored in a plain text file with two columns, separated by at least one white space character. Here, the first column specifies the nucleotide number, starting with 1, while the second column contains the corresponding normalized reactivity value. Any reactivity value below 0 will be interpreted as missing data, and thus not included in the folding recursions. Positions for which no reactivity data is available may also be left out from the table, see Figure S2A for an example.

3 Computational overhead

To assess the computational overhead induced by the implementation of the additional layer that allows for the application of hard and soft constraints, we performed a comparison of run-time and memory consumption for the program `RNAfold`. We compared the averaged computational requirements for MFE, partition function, and base pair probability computations of sets of random RNA sequences with lengths between 100 nt and $30,000$ nt. For each of them, we generated five settings, (1) default (no explicit constraints), (2) a single hard constraint that forces a particular nucleotide to stay unpaired, (3) a single nucleotide soft constraint, and (4) a soft constraint that adds a bonus energy for a particular base pair. As a reference setting, we chose (5) `RNAfold` of version 2.1.9 that does not implement the new additional constraints layer. As visible in Figure S3, there is virtually no run-time overhead of our implementation for MFE computations. Though, memory requirements grow due to the usage of the additional upper triangular matrices that we use as storage for the constraints. However, our implementation of an additional layer for the partition function and base pair probability computations does in fact increase both, running time, as well as memory requirements, although the effect on the running time is rather small. In fact, our implementation is still very efficitent, both in terms of computation time and memory consumption, and outperforms those of `Fold`

A SHAPE reactivity input file

```
9    -999       # No reactivity information
10   -999
11   0.042816   # normalized SHAPE reactivity
12   0          # also a valid SHAPE reactivity
15   0.15027    # Missing data for pos. 13-14
...
42   0.16201
```

B Constraints definition file

```
F i 0 k   [TYPE] [ORIENTATION] # Force nucleotides i...i+k-1 to be paired
F i j k   [TYPE] # Force helix of size k starting with (i,j) to be formed
P i 0 k   [TYPE] # Prohibit nucleotides i...i+k-1 to be paired
P i j k   [TYPE] # Prohibit pairs (i,j),...,(i+k-1,j-k+1)
P i-j k-l [TYPE] # Prohibit pairing between two ranges
C i 0 k   [TYPE] # Nucleotides i,...,i+k-1 must appear in context TYPE
C i j k          # Remove pairs conflicting with (i,j),...,(i+k-1,j-k+1)
E i 0 k e        # Add pseudo-energy e to nucleotides i...i+k-1
E i j k e        # Add pseudo-energy e to pairs (i,j),...,(i+k-1,j-k+1)

                 # [TYPE]        = { E, H, I, i, M, m, A }
                 # [ORIENTATION] = { U, D }
```

Figure S2: **Input file formats for the new hard/soft constraint features of the `ViennaRNA Package`.**
(**A**) Normalized SHAPE reactivities are provided in column-wise fashion, where the first column specifies the nucleotide position, and the second the actual reactivity value. Negative reactivities are treated as not available, hence do not contribute to the guided predictions. Missing data may also be indicated by just leaving the corresponding row out of the input file. (**B**) Simple nucleotide-, and base pair-wise constraints may be specified in Constraint definition files. Similar to the constraints file format used in `UNAfold/mfold`. each line starts with a single character command, followed by three or four numbers. In addition, optional auxiliary modifier characters may be used to limit the constraint to specific loop types. For base pair specific constraints, we currently distinguish pairs in exterior loops (E), closing pairs of hairpin loops (H), closing (I) and enclosed (i) pairs of interior loops, and closing (M) and enclosed (m) pairs of multibranch loops. Nucleotide-wise constraints may be limited to their loop context using the corresponding uppercase characters. The default is to apply a constraint to all (A) loop types. Furthermore, pairing constraints for single nucleotides may be limited to upstream (U), or downstream (D) orientation.

of the `RNAstructure` package, and `mfold/UNAFold` (data not shown).

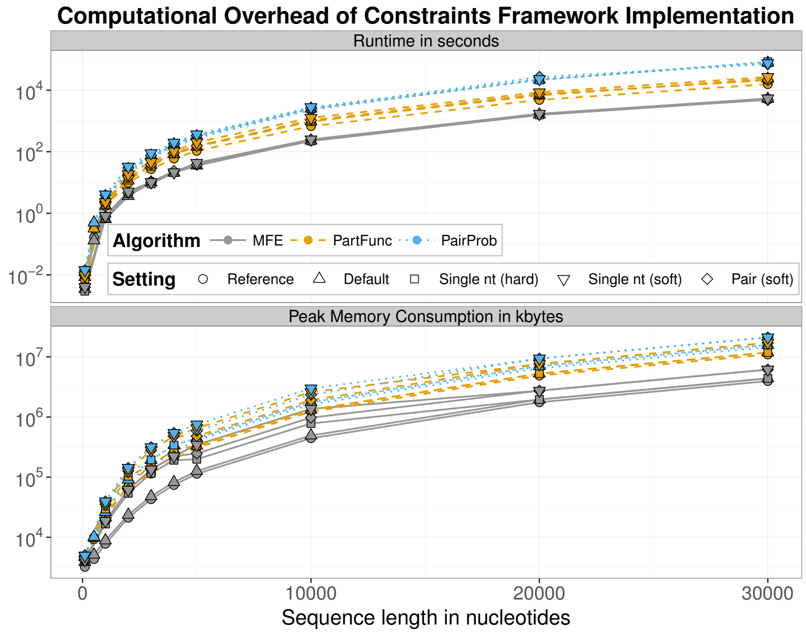

Figure S3: **The computational overhead of the additional layers that facilitate hard/soft constraint support.**
Upper panel shows averaged run-time in seconds, while the lower panel displays the peak memory consumption in kilobytes. Each panel shows five software settings where for each of them three different algorithms were run: Minimum free energy (MFE), partition function (PartFunc), and partition function with subsequent base pair probability computations (PairProb). Requirements for `RNAfold` 2.1.9 were chosen as reference setting, while the remaining four are different modes of the new `RNAfold` 2.2.0 with: (i) default settings without explicit constraints, (ii) single nucleotide hard constraint, (iii) single nucleotide soft constraint, and (iv) base pair soft constraint. Input data were random RNA sequences of length 100 (1000), 500 (100), 1000 (100), 2000 (16), 3000 (16), 4000 (16), 5000 (16), 10000 (1), 20000 (1), and 30000 (1) nucleotides, where the values in parenthesis depict the numbers of individual sequences for the particular set for which averaged requirements are derived from.

References

[1] Katherine E. Deigan, Tian W. Li, David H. Mathews, and Kevin M. Weeks. Accurate SHAPE-directed RNA structure determination. *Proc. Natl. Acad. Sci. USA*, 106:97–102, 2009.

[2] Frank Jühling, Mario Mörl, Roland K Hartmann, Mathias Sprinzl, Peter F Stadler, and Jörn Pütz. `tRNAdb` 2009: compilation of tRNA sequences and tRNA genes. *Nucleic Acids Res.*, 37:D159–D162, 2009.

[3] N R Markham and M Zuker. UNAFold: software for nucleic acid folding and hybridization. *Methods Mol Biol*, 453:3–31, 2008.
